# Sabin polio virus protein 1 (VP1) evolution in patients with acute flaccid paralysis from 2010 to 2016 in Uganda

**DOI:** 10.1186/s12985-023-02143-7

**Published:** 2023-08-02

**Authors:** Mary Bridget Nanteza, Barnabas Bakamutumaho, Phionah Tushabe, Prossy Namuwulya, Molly Birungi, Rajab Dhatemwa, James Peter Eliku, Mayi Tibanagwa, Proscovia Kakooza, Henry Bukenya, Josephine Bwogi, Charles Rutebarika Byabamazima

**Affiliations:** 1 Uganda Virus Research Institute, Plot 51-59 Nakiwogo Road, P. O. Box 49, Entebbe, Uganda; 2 WHO Country Office, P. O. Box 24578, Kampala, Uganda; 3 World Health Organization AFRO, East and Southern Africa (ESA), 82-86 Enterprise Road, Highlands, Belvedere, P. O. Box BE 773, Harare, Zimbabwe

**Keywords:** Sabin poliovirus, VP1, Mutation, Acute flaccid paralysis, Vaccine-derived polioviruses, Uganda

## Abstract

Acute flaccid paralysis (AFP) is a rare side effect of the oral polio vaccine but can be associated with outbreaks and permanent disability in patients harboring circulating vaccine-derived polioviruses (cVDPVs). With the advancement of polio abolition in a glimpse, cVDPVs are causing outbreaks and slowing the polio eradication process. The polio virus protein 1 (VP1) contains the binding site that is key for virus transmission. Understanding the evolution of VP1 among AFP patients could yield more insight into the early events of cVDPVs. Polioviruses were identified from stool specimens of AFP patients using cell culture; and confirmed by the real time RT PCR intra-typic differentiation and vaccine-derived poliovirus assays. Seventy-nine (79) Sabin-like poliovirus 1 (SL1) and 86 Sabin-like poliovirus 3 (SL3) were sequenced. The VP1 amino acid substitutions T106A in Sabin poliovirus 1 and A54V in Sabin poliovirus 3 were common among the AFP patients as has been found in previous studies. Other substitutions that were associated with AFP were: T290A and A54T in SL1 and SL3 respectively. Nucleotide mutations that were common among the AFP patients included T402C, C670A, and T816C in SL1, and G22A, C375Y, A472R, and A694T in SL3 polioviruses. Characterizing mutations that are associated with AFP could contribute to efforts pursued to mitigate the risk of vaccine-derived polioviruses and promote development of safer vaccines.

## Background

The poliovirus belongs to *Enterovirus C species* and has 3 serotypes; serotype 1, 2 and 3. Transmission occurs through the fecal–oral route. The virus multiplies in the gut and in 2% of infected persons, it enters and replicates in the nervous system resulting in muscle paralysis that manifests as acute flaccid paralysis [1]. Acute flaccid paralysis (AFP) is clinically characterized by sudden onset of weakness and fever. The patient presents with asymmetrical paralysis, reduced muscle tone however, sensation remains intact [2].

The genome of the poliovirus is 7.5 kb long with one open reading frame which is translated into four structural proteins (VP1–VP4) and non-structural viral proteins (2A–2C, 3A–3D). The VP1 protein plays a role in virus binding and has been used to type and track poliovirus transmission [3]. Furthermore, it has been shown to predict phylogenetic inference for the complete genome thus the small protein has been used to estimate the evolutionary relationship for polioviruses [4]. Virus RNA packaging and release has been mapped to the amino end of the VP1 region [5]. Lastly, the VP1 forms part of the major neutralization site that seldom contribute to virus escape [6, 7].

The evolution of VP1 of the poliovirus occurs because of the lack of 3′–5′ exonuclease proof reading mechanism for the virus coded RNA dependent RNA polymerase [8, 9]. During virus replication, the RNA polymerase introduces mutations in the virus genome, some of which occur in the VP1 region and are occasionally associated with reversion to a neuro pathogenic trait. The rate of misincorporation of bases ranges 10^−5^–10^−3^ per replication cycle [10, 11]. VP1 substitutions accumulate at a higher rate in Sabin-like polioviruses than the wild polioviruses [12] and occur within the first or second months following the administration of oral polio vaccine (OPV) [13, 14]

Numerous mutations have been identified among patients of AFP. These are usually silent synonymous mutations, and a selective constraint is applied on the non-synonymous ones [15, 16]. Yan et al. [17] identified one nucleotide mutation C161U that resulted in substitution A54V in 3 out of 4 Sabin-like type 3 polioviruses collected from a cluster of high-risk AFP patients. In another study, amino acid changes in VP1 of Sabin polioviruses 1 namely H149Y, T106A or I90L have been reported among AFP patient with residual paralysis [18].

De-attenuating mutations like T6I in Sabin poliovirus 3 [19] and single mutations can also alter pathogenesis; a single mutation (I143T) in Sabin poliovirus 2 VP1 region has been associated with neuro-pathogenesis [20]. Numerous mutations among AFP patients result in vaccine-derived polioviruses (VDPV) and are classified as VDPV type 1, 2, and 3 [21].

The aim of this study was to characterize VP1 mutations of Sabin poliovirus serotype 1 and 3 isolates that are associated with acute flaccid paralysis in Uganda. This study could provide additional insight into the early events of vaccine-derived polioviruses and could open avenues for intervention.

## Methods

It is a retrospective cross-sectional study designed to investigate mutations that are common in patients of acute flaccid paralysis. The permission to undertake this study was obtained from the Ministry of Health, Uganda, the owner of the specimens and the Uganda Virus Research Institute, Research and Ethics Committee approved the study.

Archived poliovirus isolates from AFP patients aged 1 day–180 months were identified through the routine national AFP surveillance system. The Sabin poliovirus 1 and 3 that were investigated were collected between 2010 and 2016 before the switch from trivalent OPV (OPV 1, 2, and 3) to bivalent OPV (OPV1 and 3). The incidence of AFP patients during this period ranged from 2.3 to 8.5% (data not published). The percentage described represents the number of AFP patients who were shedding the poliovirus compared to the total number of AFP patients who were reported annually. The highest rate was observed in 2016 and corresponded to the period of enhanced polio campaigns prior to the switch.

### Virus isolation

Poliovirus isolation was primarily characterized through cell culture using the rhabdomyosarcoma (RD) and mouse-derived L cell lines (L20B) [22]. The poliovirus type was then confirmed using real time Reverse Transcriptase Intra-Typic Differentiation and Vaccine-Derived Poliovirus assays (rRTPCR ITD and VDPV assays) [23].

### RNA extraction

RNA extraction was performed from archived virus isolates using QIAamp Viral RNA mini kit (Qiagen) according to the manufacturer’s protocol.

### Sabin RT-PCR procedure

The procedure was adopted and optimized using the standard protocol for VP1 gene characterization (CDC Laboratory). The Qiagen One step RT-PCR was used according to the manufacturer’s protocol. Briefly the RT-PCR amplifying a 1.1 kb encompassing the VP1 region was performed in a 50 µl reaction that consisted of: 30.5 µl RNase free water; 10.0 µl 5 × Qiagen One Step RT-PCR buffer; 2.0 µl dNTPs (10 mM each); 2.0 µl 5 × Qiagen One Step RT-PCR enzyme mix; 1.0 µl Y7R primer (40 pmol/µl), 5′GGTTTTGTGTCAGCITGYAAYGA-3′; 1.0 µl Q8 primer (10 pmol/µl), 5′AAGAGGTCTCTRTTCCACAT-3′; 0.5 µl RNase Inhibitor (40 U/µl), and 3.0 µl of the template (RNA extract). The thermo-profile condition used for the RT-PCR reactions was as follows: reverse transcription 50 °C for 30 min, RT enzyme inactivation 95 °C for 15 min; amplification: 94 °C for 30 s, 45 °C for 30 s, 72 °C for 1 min × 35 cycles; final extension 72 °C for 10 min; and finally left to hold at 4 °C.

### Gel electrophoresis

One gram (1.00gm) of agarose powder was weighed and added to 100mls of 0.5% TBE buffer which was heated and cooled. Four micro-litres (4.0 µl) of ethidium bromide were added to the gel and a comb positioned in the casting tray to create wells for the samples. After the gel had solidified, the PCR products were then loaded onto the wells. A 100 bp molecular DNA ladder marker and a positive control specimen were added for amplicon sizing to ensure that the correct virus fragment was amplified. The PCR products were run at 130 V for 45 min in the electrophoresis tank. After gel electrophoresis, the 1.1 kb DNA products were viewed under ultraviolet light and the images were taken for documentation. The DNA products were stored at 20 °C till further testing.

### Cleaning DNA

The Invitrogen Charge Switch PCR Clean-up Kit was used according to the manufacturer’s protocol. Briefly, binding of cDNA was achieved when 50 µl of the purification buffer and 50 µl PCR product together with 10 µl of the Charge Switch magnetic beads were mixed in a microcentrifuge tube and incubated at room temperature for one minute. The mixture was then placed on a Magna rack and the supernatant was removed and discarded. The complementary DNA (cDNA) bound to the beads was washed twice using 150 µl wash buffer for each sample. The sample was again removed from the rack and 50 µl of the elution buffer was added. The beads and buffer were mixed by gentle pipetting. The mixture was placed back on the Magna Rack and incubated for 1 min. The supernatant which contained the purified cDNA was collected, quantified using nanodrop, and stored at − 20 °C.

### VP1 region sequencing

The Big Dye Terminator v3.1 cycle sequencing kit was used according to the manufacturer’s protocol. Briefly, a 10.0 µl sequencing reaction was set up consisting of: 5.0 µl RNase free H20, 1.0 µl sequencing buffer × 5, 1.0 µl primer (3.2 pmol) (primarily 246S/249S/249A/Q8 for SL1 or 248S/251S/ 261A/Q8 for SL3), 2.0 µl Big dye, and 1.0 µl [cDNA] (20-40 ng). The sequencing positive control reaction consisted of: 4.5 µl dH20, 1.0 µl Seq. buffer, 1.25 µl primer M13, 2.0 µl Big dye, and 1.25 µl pGEM DNA. The following thermo-profile condition was used for the sequencing reactions: amplification 95 °C for 15 s, 42 °C for 15 s, 60 °C for 4 min for 25 cycles and left to hold at 4 °C. The DNA was then stored at − 20 °C.

### Cleaning sequencing products

Sequencing product cleanup was performed using the Agencourt ‘Clean SEQ according to the manufacturer’s protocol with a modification. Briefly, 10 µl of the magnetic beads were added to each specimen. Forty-two (42) µl of 85% ethanol was added to the specimen and mixed to form a homogeneous mixture. To enhance DNA binding the plate was left for 5 min before placing on the magnet (modification). The plate was then placed on the Magnetic Agencourt block for 5 min. The supernatant was removed, and each well was washed twice using 85% ethanol while the plate was on the magnet. The plate was left to dry for 10 min at room temperature. Thereafter, 42 µl of 0.1 mM EDTA (elution buffer) was added, and the plate was incubated at room temperature for 5 min to elute the DNA. Thirty (30) µl of the clear solution that contained the eluted DNA was carefully removed with a pipette ensuring that there was no bead carry over. The DNA product was transferred into the new wells ready for loading onto the detector. The plate was sealed with a septae and stored at − 20 °C until it was run on the 3500 ABI Genetic analyzer.

### Data analysis

The full VP1 contigs were assembled using Sequencher software 4.10.1. Alignment of the consensus sequences and phylogenetic inference were performed using Mega 7.0 sequence analysis software [24]. Conservative and non-conservative mutations in VP1 were then identified in each independent alignment. The identification of nucleotide and amino acid mutations was performed by tally when changes in nucleotide and amino acid residues were compared to the reference Sabin poliovirus 1 and 3. Mutations were captured as point nucleotide and amino acid differences in the study VP1 sequence alignments.

### Mutations from previous circulating VDPV outbreaks

Poliovirus VP1 sequences from outbreaks linked to circulating VDPVs [25] were downloaded from the National Center for Biotechnology Information (NCBI) and analyzed. Fifty-nine (59) and 24 sequences of SL1 and SL3 were available. The study VP1 mutation sites described were compared with those identified in viruses from the reported outbreaks.

### AFP surveillance system

The study specimens investigated were kindly obtained from the routine national AFP surveillance system and the data reported was obtained from the “poliomyelitis/ AFP investigation data collection tool for acute illness”. The clinical presentation, immunization history, together with the preliminary specimen tracking were obtained from the collection tool. Sabin follow-up was performed for the AFP cases in whom the Sabin-like viruses were isolated.

## Results

The VP1 sequencing protocol was primarily optimized to detect polio sequences. A total of 79 SL1 and 86 SL3 were sequenced from the archived poliovirus isolates. In total, there were 65 SL1s, 81 SL3s and 16 mixtures of SL1 and SL3 polioviruses. Two (2) SL1 and 7 SL3 sequences failed on sequencing and 4 SL3 were missing. Twenty-nine and 50 mutations were observed in Sabin poliovirus 1 and 3 respectively. The nucleotide and amino acid mutations identified are respectively shown below in Tables 1 and 2.

**Table 1:** Nucleotide and corresponding amino acid changes among Sabin polioviruses type 1 isolates

Nucleotide change	Amino acid (aa) change	Nucleotide change	Amino acid (aa) change
1. C89Y	T30?	16. G586A	V196I C
2. C103Y	P35?	17. C639T	~
3. G126R	P42?	18. C665Y	A222?
4. A138R	A46?	19. **C670A × 2**	~
5. A162G	~	20. T716C	I239T NC
6. A270R	I90?	21. T726**-**	V242?
7. C294T	~	22. C666Y	A222?
8. **A316G X2**	**T106A NC**	23. A786G	~
9. **A316R**	**T106?**	24. **T816 Cx2**	~
10. **T402C × 2**	~	25. G891R	K297?
11. A543G	~	26. T873C	~
12. T726-	V242?	27. **A868G**	**T290A NC**
13. T471C	~	28. **G870R × 2**	**T290?**
14. C555T	~	29. A876G	~
15. A580T	I194F NC		

**-**: deletion; ~ **:** no amino acid change; ?: amino acid change not assigned; NC: non-conservative amino acid change; C: conservative amino acid change; mutation in bold: mutation appearing more than once in the study sequences; and x: the frequency of the mutation

**Table 2:** Nucleotide and corresponding amino acid change among Sabin polioviruses type 3 isolates

Nucleotide change	Amino acid (aa) change	Nucleotide change	Amino acid (aa) change	Nucleotide change	Amino acid (aa) change
1.A11G	D4G NC	19. T314Y	M105?	37. C531Y	S177?
2.**G22R × 3**	E8?	20. G320A	R106H C	38. A541G	~
3.T27C	~	21. G345A	~	39. A597R	L199?
4.A30R	A10?	22. C351T	~	40. C630T	~
5.A32G	Q11?	23. G357A	~	41. T672C	~
6.A63G	~	24. C369Y	F123?	42. G681R	L227?
7.G66A	~	25. C375Y	Y125?	43.** A694W**	~
8.A68R	D23?	26. T394C	~	44. A694T	~
9. A74-	L25?	27. C402Y	F134?	45. A772G	~
10. T81Y	D27?	28. A408R	V136?	46. G847T	~
11. T93Y	S31?	29. A424G	~	47. C852T	~
12. C96T	~	30. 433–-	~	48. G858T	R286S NC
13.** G160R**	**A54?**	31. C447T	~	49. G868A	~
14.** G160A × 3**	**A54T NC**	32. **A472G × 2**	~	50. T877C	~
15.** C161T × 2**	**A54V C**	33. **A472R × 2**	~		
16.G210-	R70?	34. A480R	P160?		
17. A231G	~	35. C491Y	T164?		
18. T261C	~	36. C510Y	D170?		

**-**: deletion; ~ **:** no amino acid change; ?: amino acid change not assigned; NC: non-conservative amino acid change; C: conservative amino acid change; mutation in bold: mutation appearing more than once in the study sequences; and x: the frequency of the mutation

In this study, conservative and non-conservative changes have been described. A nucleotide substitution that changes the corresponding amino acid property in the protein is denoted non- conservative substitution (‘**NC’**), whereas a nucleotide substitution that does not change the amino acid property in the protein is denoted conservative substitution (‘**C’**) (see Tables 1 and 2 below).

There were more VP1 mutations for Sabin poliovirus 3 than Sabin poliovirus 1 (SL1). The SL1 viruses contained 29 mutations with 4 non-conservative substitution compared to SL3 that contained 50 mutations with 3 non-conservative substitutions.

A complementary presentation of VP1 nucleotide mutations for SL1 and SL3 sequences is shown in Table 3 placed after the text.

**Table 3:** Complementary presentation of VP1 nucleotide mutations for Sabin-like poliovirus 1 and 3

Sabin poliovirus 1			
*One mutation*			
Uga-10-2779	A270R	UGA-13-0250	T716C
Uga-11-0064	A138R	UGA-13-1953/4	A786G
Uga-11-0080	**T816C**	UGA-13-2304	**C670A**
Uga-11-0737/8	C555T	UGA-14- 2791	T726-
Uga-11-2157	T665Y	UGA-15-0498	A316R
Uga-12-1416	T816C	UGA-15-0934	**G870R**
Uga-11-2327	C89Y	UGA-16-1176	C103Y
Uga-12-2639	A868G	UGA-16-0457	A580T
Uga-12-2585	C639T	UGA-16-1223	T471C
Uga-16-0355	**T402C**		
*Two mutations*			
Uga-11-0083	**A316G,** A666G		
Uga-13-2055/6	T402C, C670A		
Uga-14-2957	A316G, A543G		
Uga-15-0398	C670A, G870R		
Uga-16--0356	T402C, G891R		

Sabin poliovirus 3			
*One mutation*			
Uga-11-2245/6	**G160A**	UGA-15-1156	**A694T**
Uga-12-1318	G22A	UGA-16-0627	T81Y
Uga-12-1317	**G22R**	UGA-16-0619	**A472R**
Uga-12-1341/2	G22R	UGA-16-1615/6	C351T
Uga-12-1698	A231G		
Uga-12-2630	G210**-**		
Uga-12-2640	A597R		
Uga-13-0807/8	A30R		
Uga-13-2602	**C375Y**		
Uga-14-4283/4	C873T		
Uga-15-0396	C510Y		
Uga-15-0402	T74**-**		
*Two mutations*			
Uga-11-0475	T394C, A541G	UGA-16-0355/6	T27C, G357A
Uga-12-2356	A32R, A408R	UGA-16-1723	A68R, G320R
Uga-14-2537/8	A11G, G160A		
Uga-15-0566	C491Y, A694W		
Ugaa-15-0727	G160A, G320A		
Uga-15-0457/8	G160?, C630T		
*Three mutations*			
Uga-12-1883/4	**C161T,** T261C, C447T		
Uga-14-0296	C161T, C375T, A472R		
Uga-14-1516	T672C, G847T, G868A		
Uga-16-0620	C369Y, A472G, A480R		
Uga-16-0621	T93Y, C402Y, A472G		
Uga-16-1518	C531Y, G681R, C852T		
*Four mutations*			
Uga-15-1045/6	G66A, G345A, 433–435 deletion, T877C		
Uga-16-3603/4	A63G, C96T, A424G, A772G		

UGA-YY-XXXX/XXXZ or UGA-YY-XXXX/Z is a study sample identifier for each AFP patient; UGA: represent the country of Uganda, YY: year of sample receipt, XXXX—odd study number for the first specimen; XXXZ—the consecutive even study number for the second specimen; **-**: deletion; and mutation site in bold: a mutation site appearing more than once in the study sequences. NB. Some AFP patients provided one specimen other than the 2 specimens that are required routinely and for others the virus was only detected in one specimen i.e., such specimens are represented by one study identification number

A total of 24 SL1 and 33 SL3 VP1 mutants have been identified in Table 3. The frequency of the nucleotide mutations for SL1 and SL3 can also be deduced from Table 3. The common nucleotide mutations sites (appearing more than once) in SLI were: A316R/G, T402C, C670A, T816C, and G870R, and those in SL3 were : G22A/R, G160A, C161T, C375Y, A472R, and A694T/W. The estimated VP1 mutation rate for SL1 and SL3 viruses in this study has been 4.1 × 10^−4^ (29 × 100/79 × 906), and 5.2 × 10^−4^ (50 × 100/86 × 900) respectively.

**Fig. 1:**
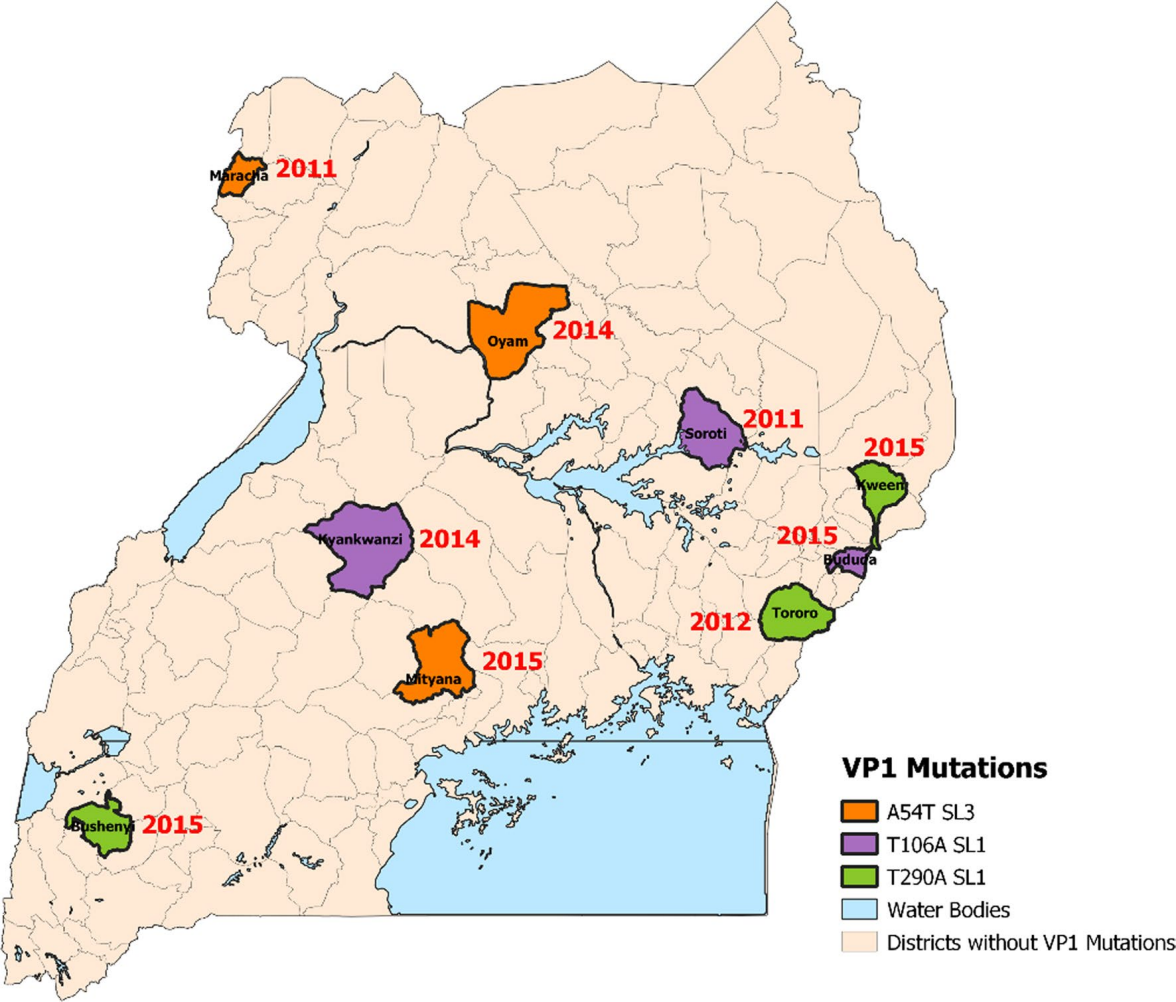
Map of Uganda showing the districts of origin of the VP1 non-conservative mutations among AFP patients

A map of Uganda showing the districts of origin for the VP1 non-conservative mutations among AFP patients is shown in Fig. 1 below.

The non-conservative VP1 mutants were identified in districts across the country at specified times. To understand VP1 sequence variation better, phylogeny inference to reference Sabin polioviruses 1 and 3 was performed (data not shown). There was minimal nucleotide divergence among the Sabin 1 and 3 sequences however, isolate UGA-16-3603/4 was divergent from the reference Sabin poliovirus 3. Study VP1 mutation sites described were compared with those identified in viruses from the reported cVDPV outbreaks. Mutation T106A in SL1 viruses; and A54V and M105T in SL3 viruses existed in the VDPVs as well [25, 26].

Table 4 displaying the relationship of the OPV dose to the mutations identified in SL1 and SL3 is shown below.

**Table 4:** Oral polio vaccine doses corresponding to VP1 mutations with complete vaccination, clinical and specimen documentation

Specimen identifier	Age in months	Date of last OPV	OPV dose	Number of OPV doses	Date of onset	Date of Stool receipt	District of origin	Number of VP1mutations
*Sabin 1 polioviruses*								
UGA-11-0737/8	28	15May2011	OPV-3	4	16May2011	23May2011	Iganga	1
UGA-12-1416	27	25May2012	OPV-3	4	21Jun2012	22Jun2012	Wakiso	1
UGA-13-2055/6	6	13Aug2013	OPV-1	2	15Aug2013	13Aug2012	Gulu*	2
UGA-13-2303/4	4	02Sep2013	OPV-3	4	05Sep2013	12Sep2013	Arua	1
UGA-16-0457	13	23Jan2016	OPV-3	4	30Jan2016	03Feb2016	Buvuma	1
Sabin 3 polioviruses								
UGA-12-1883/4	3	27Jul2012	OPV-0	1	27Jul2012	01Aug2012	Iganga*	3
UGA-14-1516	3	05Mar2014	OPV-2	3	31Mar2014	08Apr2014	Kaberamaido	3
UGA-15-1045/6	30	18Jan2015	OPV-2	3	09Feb2015	25Feb2015	Lira*	4
UGA-16-3603/4	2	13Jul2016	OPV-1	2	14Jul2016	18Jul2016	Bududa	4

OPVx; x stands for the OPV dose number according to the national immunization schedule (e.g., in Uganda OPV0 is given at birth; OPV1 given at 6 weeks; OPV2 given at 10 weeks; and OPV3 given at 14 weeks); and *: represents districts of origin for the children who had not fulfilled the routine OPV doses

Sixty-seven percent (67%) of AFP patients selected in Table 4 above had received adequate OPV doses. Acute flaccid paralysis was linked to multiple VP1 mutations in SL3 compared to SL1 isolates. The children who have been investigated above recovered with no residual paralysis.

### The relationship between the ‘time interval from vaccination to specimen collection’, and  ‘VP1 mutations’

The Mann–Whitney test was used to establish the relationship between the ‘interval from vaccination to sample collection,’ and ‘VP1 mutations’. The assessment was constrained by the missing data; the vaccination date was missing for many children. Two samples are expected per child; to avoid the bias of repeated measure, one sample was considered per child and 42% of the samples were analyzed. There was no difference in the ‘vaccination interval’ for children who had a mutation (s) and those who did not have (*p*-value 0.867).

## Discussion

There was no vaccine-derived poliovirus nor wild type poliovirus detected among specimens that were collected from 2010 to 2016. No poliovirus type 2 was identified because virus sequencing was performed after the switch from trivalent to bivalent OPV when work on type 2 polioviruses was ceased under the WHO Global Action Plan (GAP III) containment. All polio virus type 2 isolates were destroyed and only SL1 and SL3 isolates were investigated. There were 29 mutations versus 4 non-conservative mutations for Sabin poliovirus 1 and 50 mutations versus 3 non-conservative mutations for Sabin poliovirus 3 which implicates a robust negative selection for Sabin poliovirus 3. Several amino acid substitutions were common among the SL1 and SL3 isolates and these included T106A, T290A, A54V and A54T.

For SL1, T106A was identified in 3 isolates from children residing in different districts namely Soroti, Kyankwanzi and Bududa in 2011, 2014, and 2015 respectively. Kyankwazi and Bududa are 530 km apart and had an 8 months’ time interval of virus detection. The distance and time interval of processing the samples did not predict a possible epidemiological linkage. Mutation T290A in SL1 was also reported in three AFP patients from Tororo district in 2012; and Kween and Bushenyi districts in 2015. Bushenyi and Kween are 631 km apart and sample processing time interval for the two viruses was 1 month. There was a study limitation in that samples from other countries that were processed around the same time of identifying the mutation could not be included and sequenced due to ethical issues.

Another mutation A54V was identified in SL3 and was detected in 3 isolates from Soroti, Iganga, and Rubirizi districts. Isolates from Soroti and Iganga were identified in 2012 whereas the isolate from Rubirizi was identified in 2014. The specimens identified in the same year had a 5 months’ difference between the processing time. Again, the time interval was not agreeable to an epidemiological linkage. Another mutation was detected at the same position A54T. This mutation was detected in three AFP patients from Maracha, Oyam, and Mityana districts in 2011, 2014, and 2015 as well. Oyam district is 320 km distant from Mityana district and the 2 viruses from both districts were detected within a time interval of 8 months. VP1 amino acid substitution at position 106 for SL1, and 54 for the SL3 viruses existed in both AFP and reported VDPV sequences. Mutations at these sites might be linked to vaccine-derived polioviruses.

The virus sequence UGA-16-3603/4 was obtained from a 2 months’ old child who developed AFP one day after receiving OPV1. The detected isolate contained 4 mutations and was divergent from the SL3 VP1 reference sequence. The virus is unlikely to have evolved in the recipient within one day after receiving the vaccine. There are two possible scenarios; the mutant could have evolved from the first OPV dose or, it could have been a case of environmental spread. The child resided in Bududa district which had sub-optimal tOPV + 3 coverage during the study period [27]. This district is one of the hard-to-reach districts with natural calamities of landslides.

It would be interesting to know whether there are immune deficient children among the AFP children who were shedding the virus. It is not possible to identify such children in this study because specimens for AFP surveillance are obtained at two time points within an interval of 24–48 h.

Overall, the AFP patients from the different regions of Uganda did not progress to vaccine-derived polioviruses implying that the immunity of the population was adequate and able to interrupt transmission. The national OPV3+ coverage ranged from 80 to 90% during the study period [28]. Polio campaigns were conducted in the whole country in 2012, 2015, and 2016 and in addition, targeted campaigns were also performed in the high-risk regions for VDPV and wild poliovirus transmission.

## Conclusion

Several VP1 mutations common among the AFP patients have been described and supported by this study. The data generated could support innovations to design safer attenuated vaccines.

### Caveats

In this study, virus isolates were used, and culture adapted viruses might pose a bias. It was not possible to adequately relate ‘OPV doses’ and ‘vaccination interval’ to ‘VP1 mutations’ because of a gap in documentation.

## Data Availability

All data generated during this study appears in the published article.

## Abbreviations

VP1: Virus protein 1
AFP: Acute flaccid paralysis
VDPV: Vaccine-derived poliovirus
SL1: Sabin-like poliovirus 1
SL3: Sabin-like poliovirus 3
RNA: Ribonucleic acid
OPV: Oral polio vaccine
RD: Rhabdomyosarcoma cells
L20B: Mouse L cells specific for poliovirus
PCR: Polymerase chain reaction
rRT PCR: Real-time reverse transcriptase PCR
ITD: Intra-typic differentiation
CDC: Centers for disease control and prevention
TBE: Tris-Borate-EDTA
DNA: Deoxyribonucleic acid
cDNA: Complementary DNA
EDTA: Ethylenediamine tetra acetic acid
C: Conservative amino acid substitution
NC: Non-conservative amino acid substitution
NCBI: National center for biotechnology information
WHO: World Health Organization
GAP III: Global action plan III
